# Combination of Dehydration and Expeller as a Novel Methodology for the Production of Olive Oil

**DOI:** 10.3390/molecules28196953

**Published:** 2023-10-06

**Authors:** Assamae Chabni, Luis Vázquez, Celia Bañares, Carlos F. Torres

**Affiliations:** 1 Department of Production and Characterization of Novel Foods, Institute of Food Science Research (CIAL, CSIC-UAM), C/Nicolas Cabrera 9, Cantoblanco Campus, Autonomous University of Madrid, 28049 Madrid, Spain; assamae.chabni@uam.es (A.C.); luis.vazquez@uam.es (L.V.); celia.bannares@uam.es (C.B.); 2 Department of Applied Physical Chemistry, Departmental Section of Food Sciences, Faculty of Science, Autonomous University of Madrid, 28049 Madrid, Spain

**Keywords:** Alouana oil, dehydration process, expeller extraction, olive oil, water influence

## Abstract

An alternative olive oil (OO) production process has been developed based on the combination of olive dehydration, followed by extraction with an expeller press. This procedure eliminates the utilization of water and avoids the malaxation stage. Hence, no water residues are generated. In this study, the mentioned alternative methodology was compared to conventional extraction methods. High extraction yields and oil recovery were obtained with our novel procedure. On the contrary, substantial percentages of by-products were generated with conventional methodology. The quality indexes (acidity and peroxide values) of the oils obtained by the combination of dehydration and expeller (dOO) were 0.4% of oleic acid and 3 meq O_2_/kg of oil, respectively. Furthermore, none of the applied processes affected the resulting OO’s fatty acid composition and lipid profile. Total phenolic content was up to four times higher for dOO than for other olive oils and it showed resistance to oxidation with an oxidative stability index about five times higher than that for conventional olive oils.

## 1. Introduction

Olive oil (OO) and table olives are the main products of the olive tree (Olea Europaea). For a long time, olive oil has been a subject of study and research to examine both its nutritional and medicinal properties [1]. These healthy properties are related to its unique composition, which is formed mainly by triglycerides (98–99%) containing a high proportion of unsaturated fatty acids (60–85%), mainly comprised of oleic acid (55–85%) [2]. Regarding minor fraction, it consists of 1–2% of the composition comprising more than 230 chemical compounds [3], most of which are simple phenolic compounds, such as hydroxytyrosol, secoiridoids phenolics, (e.g., oleuropein and aglycone of ligstroside), and lignans [4]. All these compounds possess physiological health benefits, including strong antioxidant, anti-inflammatory, and antibacterial activities [2]. It is worth highlighting the presence of α-tocopherol (vitamin E), which plays an important role in the antioxidant activity of olive oil. The concentration of all these compounds depends on several factors, such as harvesting time, olive variety, and extraction conditions [5].

Regarding harvesting time, as the maturity of the olive increases, the quantity of total phenolic and o-diphenols, which are responsible for the oil bitterness, decreases [6,7]. As for the olive variety, it has been observed that the content of phenolic compounds varies [6,8] being higher in those varieties with late maturation [7]. In particular, the present study focuses on the Moroccan Picholine olive variety, which is characterized by its high adaptability to temperature fluctuations and its oil presents a high content of phenolic compounds, vitamin E, and monounsaturated fatty acids (MUFAs) [9].

In terms of extraction conditions, one of the processes that produces a higher quality olive oil and does not require refining is the traditional discontinuous pressing process or conventional extraction (CE). This procedure involves several stages: a first stage of crushing the olives with a millstone crusher. Then, a malaxation phase is carried out, which consists of mixing the crushed olives with water (15–20%) until an olive paste is formed. The oil is separated from the olive paste with a hydraulic press and recovered by decantation or vertical centrifugation. After crushing the olives and mixing, separation is carried out by horizontal centrifugation (decanter) [10].

In all these extraction processes, the use of water is necessary to recover most of the oil contained in the olive during processing (usually 50–70% of the initial olive weight). However, the addition of water to the olive paste produces negative effects on the quality indices of the OO, significantly decreases its total phenolic content, and negatively affects oxidative stability [11], in addition to the generation of a large amount of olive oil by-products [12].

Therefore, emerging methods have been proposed to assist the processing of olives by reducing the use of water and other coadjuvants during the malaxation process, such as hydrated magnesium [13], calcium carbonate, or sodium chloride [14]. Some of these methods, which are directly applied to the paste, are pulsed electric fields, microwaves, and low and high-frequency ultrasounds [15,16]. In addition to increasing the extraction yield, these methods provide thermal and/or mechanical effects that damage the cell wall and hence facilitate the extraction avoiding the use of organic solvents. The effects of these extraction methods on the extractability and quality of the oil have received little attention from the industries, probably due to the associated increase in production costs [15]. However, all these procedures increase the processing steps, require the addition of water, and maintain the generation of aqueous by-products.

Alternatively, OO can be obtained from roasted olives, which eliminates the water content of the olives, followed by expeller press extraction. This is a local and unique methodology carried out in the Rif region of Morocco. The process is typically used for the extraction of Alouana oil, a unique type of olive oil characterized by its particular smoky taste, which has been made for centuries from roasted olives [17]. Similarly to roasted, different dehydration processes can be utilized to remove the water content from olives (vacuum drying, drum dryer, microwave drying, or lyophilization) [18]. The dehydration process allows the olives to be preserved for a longer time before their processing, which guarantees the complete elimination of fungi and avoids the hydrolytic rancidity of the olives because one of the main problems in the OO industry is the fusty defect of the olives during storage [19].

Particularly interesting is the expeller press, a commonly used physical method for extracting oil from seeds and nuts. It has been previously described as a highly efficient methodology in the extraction of olive pomace oil. Hence, it can be considered a sustainable and environmentally respectful method [20]. Dry materials are fed into a hopper and then compressed by a rotating screw, resulting in the extraction of oil. The process is aided by electric resistances that heat up the raw materials, making the oil less viscous and easier to extract. Once the oil is extracted, it is expelled through a hole at the bottom of the cylinder. The by-product is then removed through a nozzle located at the end of the screw. This process yields two products: oil and press cake [21].

Consequently, the combination of the dehydration process and the expeller extraction avoids the use of water in the olive oil industry, reducing the production of olive mill wastewater (OMWW) and increasing the OO quality. All this results in an efficient, clean, and environmentally sustainable extraction process.

The main aim of the present work is to compare different conventional OO extraction processes with various hydration degrees, and a methodology based on the combination of dehydration and expeller. To evaluate the oil obtained, different quality parameters have been studied (peroxide, p-anisidine, and acidity values). Also, fatty acid and lipid profiles, total phenolic compounds, and the resistance against oxidation by the measurement of the oxidative stability index at 120 °C have been carried out for all the oils produced.

## 2. Results and Discussion

### 2.1. A Comparative Study of Different Extraction Procedures

The moisture content, total fat content, and the parameters obtained from the different extractions are shown in Table 1. Moisture and total fat contents of raw olives (56.7 ± 1.3% and 21.5 ± 2.6%, respectively) were consistent with data reported for Moroccan Picholine and other Mediterranean varieties cultivated in a range of environments and harvest times (49–60% for water content and 20–25% of total fat content) [22]. It should be noted that the initial moisture content of the olives should be considered before processing since it is going to have an enormous influence on the amount of water to be added [11]. As can be seen in Table 1, the addition of 5% (*w*/*w*) of water to the olive paste (OO) does not show differences with the extraction without added water (OO_ww_) in terms of extraction yield, oil recovery, and material balance. These results are consistent with those obtained by Novoselic et al. [23] in a study comparing the same quantities of water added. This may be due to the high initial moisture of the olives (56.7%) since the addition of water is commonly used when the moisture of the olives to be processed is low [24,25].

Regarding the expeller extractions (dOO_1–3_), the water content of the sample to be processed plays an important role. An increase in humidity causes a change in the plastic properties of the olive, acting as a lubricant, reducing the level of compression during extraction and, consequently, decreasing the oil recovery [21].

As shown in Table 1, when the moisture content is higher, the extraction yield and oil recovery decrease. The best extraction yield obtained is 34.4% (dOO_1_), which represents 12% in the non-dehydrated olive. This value is close to that obtained with conventional extraction (CE), although without generating aqueous residues (OMWW). With the expeller extraction, press cake does not need any additional stabilization procedure since it has a moisture content of less than 2%. This is an important advantage compared to the olive pomace obtained by the traditional method, which usually contains 30% humidity in addition to the OMWW generated (which represents 40–60%; *w*/*w*).

### 2.2. Quality and Oxidation Indices

**Figure 1:**
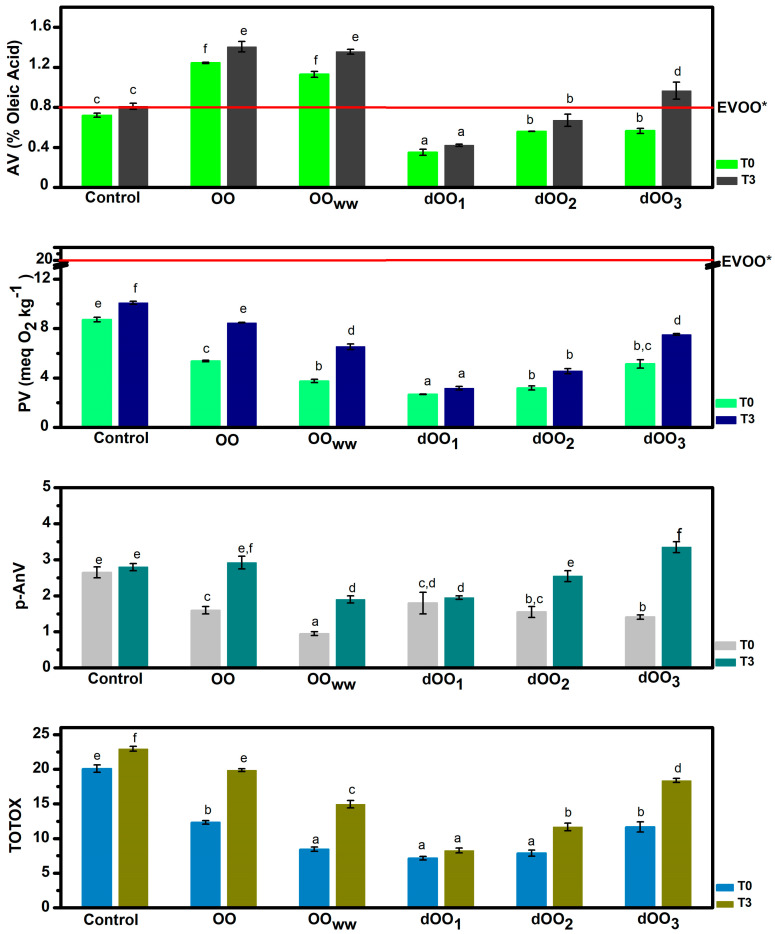
Acidity value (AV), peroxide value (PV), p-anisidine value (p-AnV), and total oxidative value (TOTOX) of obtained OO samples at the initial time (T0) and after three months (T3). Data are expressed as mean ± standard deviation. Mean values with different superscript letters are significantly different (*p* < 0.05). * EVOO: extra virgin olive oil quality criteria, value limits set by European Union.

The data depicted in Figure 1 show the results of acidity, peroxide, p-anisidine, and TOTOX values of olive oils at time zero and after 3 months of storage in the dark under an inert atmosphere (N_2_) at room temperature. At the initial time (t_0_), the quality parameters of olive oils obtained in the present study (acidity and peroxide value) remain below the limits established by the International Olive Council (IOC) and the European Regulation for extra virgin olive oil (0.8% oleic acid and 20 meq O_2_/kg oil), except for the oils extracted from raw olives (OO and OO_WW_) (Figure 1), whose acidity is higher. This may happen because olives have been damaged during transport and consequently intrinsic lipases may have caused some triglyceride hydrolysis. Moreover, due to their higher moisture content, olives become more susceptible to pathogenic infections and mechanical damage during storage [28]. However, this does not occur in any of the oils obtained from dehydrated olives with expeller (dOO_1–3_), since no significant differences were observed (*p* > 0.05). Since fungi are eliminated in this case, bacterial growth does not occur and the consequent hydrolytic rancidity of the olives is avoided [29]. 

During the three months of storage, no significant variation in acidity was observed (*p* > 0.05). Except for dOO_3_, extracted by expeller from an olive with 20.4% moisture, part of this moisture could have remained in the oil and accelerated the hydrolysis rate.

The peroxide value (PV) is the most representative parameter considered to measure primary oxidation in olive oils [30]. Initially, the PV of oils extracted from dehydrated olives (dOO_1_ and dOO_2_) and those extracted without water (OO_WW_) is below 6 meq O_2_/kg oil (Figure 1), being significantly different from those extracted by adding water (OO) and those partially dehydrated (dOO_3_). This is to be expected in oil extracted from raw olives (OO_WW_). Some studies have reported similar results in which the addition of water during malaxation has been shown to produce a negative effect on the PV since water addition enhances undesirable primary oxidation reactions [11,24,31]. As for oils extracted from dehydrated olives with moisture lower than 10% (dOO_1_–dOO_3_), the low PV is probably caused by the fact that heating the olives can lead to the accelerated degradation of hydroperoxides toward the formation of secondary oxidation products [32].

In the three months of storage, the variation in PV was significant (*p* = 0.027 for control oil, *p* = 0.4 × 10^−5^ for OO, *p* = 0.008 for OO_WW_, and *p* = 0.0204 for dOO_3_) for all the oils from olives whose processing moisture content was greater than 10%. This variation may be caused by the fact that water plays an important role in the partition coefficient of the minor compounds between oil and water, reducing their concentration in oil. Phenolic compounds have an amphiphilic character and therefore they have a certain affinity to the aqueous environment [11]. This phenomenon reduces the number of antioxidants in the oil that should protect it against oxidation during storage. On the contrary, oils extracted by expeller from dehydrated olives (dOO_1_ and dOO_2_) do not show significant differences (*p* = 0.08 and 0.054, respectively) between the initial time and three months after storage.

The determination of the p-anisidine value (AnV) is carried out using an empirical test (see Section 3.2.4 for details) that estimates the secondary oxidation products of unsaturated fatty acids, mainly consisting of conjugated dienals and 2-alkenals. AnV is particularly useful for evaluating the quality of frying oils [33], but surprisingly, it is not contemplated in the food industry for olive oils, so it has no established limits. Initially, there were no significant differences between the AnV values (AnV < 2) of the oils extracted from raw olives (OO and OO_WW_) and those extracted by expeller of dehydrated olives (dOO_1_–dOO_3_). These AnV results are even lower than those obtained by Giuffrè et al. [34] for the EVOO (4.4 ± 0.1 AnV). After three months of storage, secondary oxidation reactions had increased in all oils except the control oil and dOO_1_, probably due to an increase in the number of compounds caused by hydroperoxides degradation and moisture, as Méndez and Falqué [35] verified in their study on the effect of the storage time on quality parameters of extra-virgin olive oil.

TOTOX is an indicator of the overall oxidation state and quality of the oil [36]. It is a combination of the PV, as a measure of the quantity of primary oxidation products (hydroperoxides), and the AnV, as a measure of the secondary products (mainly alkenals and alkadienals), in fats or oils. Thus, the higher the TOTOX value, the higher the total oil oxidation. TOTOX values of the oils in the present work are shown in Figure 1. There is no established limit of TOTOX values for vegetable oils. As a reference, we use the limit for oils from marine sources. Oils with TOTOX values below 26 are suitable for human consumption. [37]. All the oils tested in this study had TOTOX values below this limit.

### 2.3. Fatty Acid Profile of Oils

Table 2 shows the percentage of fatty acids (FAs) detected in control and extracted olive oils. It should be mentioned that the fatty acid composition of vegetable oils has a considerable influence on their stability and nutritional value [26,38]. Identified FAs were mostly oleic acid (C18:1 n-9) (65–78%), followed by palmitic acid (C16:0) (8–17%), linoleic acid (C18:2) (6–12%), and stearic acid (C18:0) (2–2.8%). The amounts of α- and γ-linolenic acids were 0.8–1.36 and 0.3–0.45% of the FAs, respectively, whereas mass balance containing other fatty acids was less than 2.7%. The FA composition (%) of samples (Table 2) is within the limits established for extra virgin olive oil (EVOO) by the European Union [39] with MUFAs as the larger portion of FAs as expected. These values were consistent with the published data for the Moroccan Picholine variety [9,21,26]. Almost no significant differences in the FA composition were observed depending on the extraction method used. Therefore, the amount of water added during malaxation does not influence the composition of FAs, as confirmed by other authors [40], nor does olive roasting, thereby confirming the good stability of olive oil FAs [26,32]. 

The oleic/linoleic acid ratio (O/L ratio) is relevant for the effects on nutritional properties and oxidative stability of olive oils [41]. Regarding the O/L ratio obtained in this work, the results agree with those obtained by Mansouri et al. [42] for picholine variety oils. When the O/L ratio is low, which is related to low oleic acid content in terms of linoleic acid [43], the stability of the oil and, therefore, its shelf life, could be affected [38].

### 2.4. Lipid Profile of Oils

**Figure 2:**
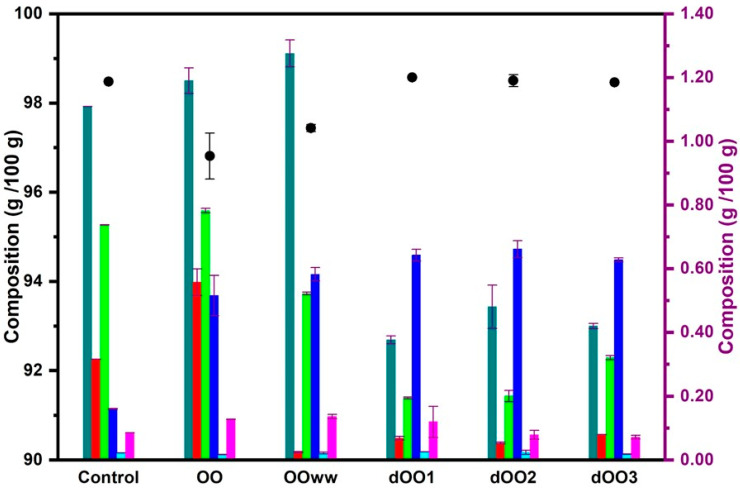
Lipid composition (%) for control oil and the oils obtained via centrifugation (OO and OO_WW_) and expeller extraction systems (dOO_1–3_). Left axis (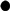 Triacylglycerol), right axis (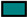 diacylglycerols, 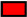 monoacylglycerols, 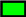 fatty acids, 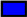 squalene, 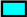 α-tocopherol, and 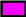 sterol). Data are expressed as mean ± standard deviation.

The results of the analysis of the lipid profile of the oils are shown in Figure 2. Significant differences (*p* < 0.05) in TAG content were observed between oils from raw olives (OO and OO_WW_) and those obtained with expeller from dehydrated olives (dOO_1_–dOO_3_). This is probably caused by the fact that the initial moisture content of the olives favors TAG hydrolysis during storage [28,44], reducing the TAG content in the oil and increasing the DAG, MAG, and FFA content, as can be observed in Figure 2. The amount of TAG obtained for OO and OO_WW_ (96–98%), although it is low, agrees with that obtained by Cossignani et al. [45] confirming that the higher the fatty acid content, the higher the DAG content observed [44]. Regarding dOO_1_–dOO_3_, TAG content was not altered by the olive heating and the expeller extraction process. The results obtained (98–99%) were similar to those obtained by other authors for EVOO (≈99%) [2,44].

The composition of minor compounds in olive oils is conditioned by factors such as the olive variety, harvest time, applied method for oil extraction, and storage conditions [2]. As for the identified minor compounds (0.8–2%), the high content of DAG, MAG, and FFA in the oils is due to the TAG hydrolysis. The content of other minor compounds such as squalene, tocopherols, and sterols, was not affected by the extraction process applied (centrifugation with and without water and expeller) or, surprisingly, the prior dehydration of the olives. The results obtained for the studied oils (OO, OO_WW_, and dOO_1_–dOO_3_) for squalene (3000–7000 mg/kg of oil), sterols (600–1500 mg/kg of oil), and α-tocopherols (170–250 mg/kg of oil) show no significant differences (*p* < 0.05) between the oils and were consistent with those obtained by other authors for olive oil of the Moroccan Picholine variety, both for conventional olive oils [9,26] and for oils from roasted olives [32]. Furthermore, they are within the limits reported for EVOO [2,46].

### 2.5. Total Phenolic Compounds and Oxidative Stability 

**Figure 3:**
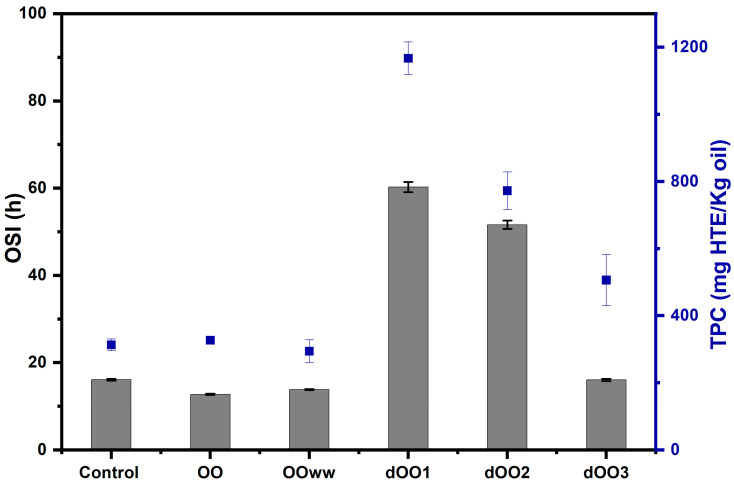
Relation between the Oil Stability Index (OSI) at 120 °C (grey bars) and the total phenolic content (TPC, blue dots) of the obtained OO samples. Data are expressed as mean ± standard deviation.

The phenolic fraction of olive oil contains over 36 structurally distinct phenolic substances (phenolic acids, flavonoids, lignans, secoiridoids, etc.). Despite the high concentration of phenolic compounds in olive fruit (3000–19,500 mg/kg), only about 2% are transferred to olive oil during the extraction procedure [2,5,31]. The total phenolic compounds (TPC), expressed as milligrams of hydroxytyrosol equivalent (HTE) per kilogram of oil for the obtained oils are shown in Figure 3. As for the oils extracted from crude olives (OO and OO_WW_), the results obtained (327 and 294 mg of HTE/kg of oil, respectively) agree with those obtained by other authors for crude olive oils of the Moroccan picholine variety (200–400 mg of gallic acid/kg of oil) [47]. Furthermore, the addition of 5% (*w*/*w*) of water during malaxation did not influence the TPC content, since no significant differences were observed, in agreement with the results obtained by Novoselic et al. [22] and Kiritsakis et al. [31]. Other authors have reported significant differences in TPC by adding 1.2 and 3.5% water to the Arbequina variety olive paste [24]. Beltrán et al. [48] pointed out that if the water dose is increased, then TPC is reduced [11,23]. 

As for the oils extracted by expeller from dehydrated olives (dOO), there were significant differences in the TPC concerning OO, OO_WW_, and control, being up to four times larger (Figure 3). This is probably due to the fact that phenolic compounds remain in the olives after their dehydration, and thus most of them could be extracted from the oil because there is no water to retain them [11]. As the processing humidity increases, dOO_3_ > dOO_2_ > dOO_1_, lower TPC is found (Figure 3), being 1167 ± 48 mg of HTE/kg of oil for dOO_1_, followed by dOO_2_ and dOO_3_, with values of 772 ± 55 and 506 ± 75 mg of HTE/kg of oil, respectively. 

The oil stability index (OSI) measured for the oils by using the Rancimat test at 120 °C and airflow of 15 L h^−1^ is also shown in Figure 3. The control oil, OO, and OO_WW_ had OSI values of 16, 12.7, and 13.5 h, respectively, which are similar to those obtained by other authors for EVOO extracted from Moroccan Picholine olive varieties, achieving OSI values of 8 to 18 h [24,48,49]. As can be observed, oils extracted by the expeller from dehydrated olives (dOO_1_ and dOO_3_) had 1.5 to 4.7 times longer OSI values than OO, OO_WW_, and the control oil. The presence of water also had a negative impact on the oxidative stability of the extracted oils, as other authors have reported [24], and as in the case of dOO_3_ (16.1 h), OO_WW_, and OO, which are extracted from olives with 20, 56.7, and 61.7% of water content, respectively.

Oxidation resistance is attributed to the fatty acid profile, mainly MUFAs that transfer stability to the olive oil, and the content of minor compounds with high antioxidant activity such as tocopherols, sterols, and phenolic compounds [49]. Regarding phenolic components, a similar trend has been found between the OSI and TPC values, which indicate a direct relationship between the polyphenols content and oxidative stability; i.e., the higher the TPC content, the higher the OSI value of the oils. This can be seen in Figure 3 and agrees with the work of other authors [49,50]. 

Finally, the stability of oils (OSI) extracted with an expeller and whose processing humidity was less than 10% (dOO_1_ and dOO_2_) were 60.2 and 51.6 h, respectively. The reduction of water content from olives and the elimination of the malaxation process prior to extraction made it possible to obtain high-quality oils with a high content of minor compounds and, consequently, high OSI values. An optimization of dehydration and expeller design should be relevant for industrial applications according to the results obtained in this study.

## 3. Materials and Methods

### 3.1. Materials

The olives were Moroccan Picholine variety produced during the first half of the 2019/2020 crop season from the commune of Bni Abdellah, province of Al Hoceima (Morocco). The methodologies followed in this study require a control oil, and for this aim, a commercial extra virgin olive oil from the same variety extracted by a traditional oil mill with a hydraulic press has been used. Solvents used were hexane (HEX), methyl-tertbutyl ether (MTBE), isopropyl alcohol (ISOP), methanol (MeOH), and chloroform (CLF), which were supplied by Macron (Avantor Performance Material, Center Valley, PA, USA). Reagents used for determinations as phenolphthalein, Folin–Ciocalteu reagent and sodium carbonate (Panreac, Barcelona, Spain), boron trifluoride methanol complex solution (BF3) 14% in methanol from Sigma-Aldrich (St. Louis, MO, USA), and hydroxytyrosol (HT) standard ≥ 98% were supplied by Seprox Biotech (Madrid, Spain).

The reagents used in chromatographic analyses were pure standards of oleic acid ≥ 99%, monolein ≥ 90.9%, and 1,3 diolein ≥ 99%. Squalene ≥ 98% and α-tocopherol ≥ 98% were supplied by Sigma-Aldrich (St. Louis, MO, USA), and sterol ≥ 99% were supplied by Vita-Solar Biotechnology Co., Ltd. (Xi’an, China). 

### 3.2. Methods 

#### 3.2.1. Moisture Determination

The total moisture of the olives before and after the addition of water for processing was determined by gravimetric after drying 100 g of olives in an oven at 105 °C until constant weight using a precision analytical balance (±0.1 mg). 

#### 3.2.2. Olive Oil Extraction

Different types of olive oils have been extracted from the different batches of olives:

(a) Olive oil extracted with water (OO) and without water (OOww). The extraction method for these oils was a pilot-scale adaptation of the conventional olive oil extraction process used by the industry [13]. Approximately 5 kg of clean and raw olive fruit were crushed in a hammer mill of a small-scale olive oil machine (Ferrari Tractors CIE, Gridley, CA, USA). The crushed olives fell into a mixer and the malaxation process was carried out for 30 min. Water was added to the paste during malaxation at selected amounts (0 or 5% of added water). The malaxed olive paste (with and without water) was removed from the machine and centrifuged for 10 min at 6000 rpm (Centrifuge Sorvall LYNX 6000, Thermo Scientific, Waltham, MA, USA) and at 27 °C. Oil was removed, weighed, and stored under an inert atmosphere (N_2_) at room temperature in a dark room.

(b) Dehydrated olive oil (dOO). The clean olives were dehydrated in a ventilated oven (Memmert 600, Memmert GmbH + Co. KG, Schwabach, Germany) at 105 °C for 2.5, 3.5, and 4.5 h. This way olives were partially dehydrated and different batches of olives at different moistures were obtained. Once the bone was removed with an olive pitter (Westmark, Germany), the extraction of dOO was carried out with 250 g of dehydrated and pitted olives in an oil expeller for domestic use (Wartmann^®^, brand model WM-OP-1402A, Tilburg, The Netherlands). After the pressing process, filtered oil and press cake were obtained. The oils were stored in amber glass vials under an inert atmosphere (N_2_) at room temperature to avoid premature oxidation. The press cake was vacuum-packed and stored under refrigeration at 4 °C. The oils obtained following this process were named dOO_1_, dOO_2_, and dOO_3_, corresponding to 4.5, 3.5, and 2.5 h of the dehydration process, respectively. Oil extractions were performed in duplicate, except for dOO_3_, which was carried out once. However, in all cases, there was enough oil to conduct the analysis by duplicate.

The extraction yield (1), oil recovery (2), and material balance (3) were calculated to evaluate the efficiency of the processes and the losses as follows:

Extraction Yield (%) = (Oil weight obtained)/(Initial sample weight) × 100(1)

Oil Recovery (%) = (Oil weight obtained)/Total fat content of raw or dehydrated olives × 100(2)

Material balance (%) = (Oil weight + press cake weight)/(Initial sample weight) × 100(3)

#### 3.2.3. Total Fat Content

The total fat content of raw olives and dehydrated and pitted olives was determined using the method developed by Folch et al. [51], with some modifications. Briefly, 200 mL of CLF:MeOH (2:1 *v*/*v*) were added to 10 g of pitted olives and mixed with an Ultraturrax T18 basic IKA (Staufen, Germany) at 11,800 and 16,200 rpm for 2 min, until a homogeneous mixture was obtained, which was maintained via magnetic stirring at 900 rpm for 60 min. The sample was then vacuum-filtered and distilled water was added to the organic phase in a 1:5 ratio. The mixture was mixed in a vortex for 1 min and centrifuged for 5 min at 10,000 rpm. The lower phases were collected and placed in a decanter for 3 h. Later, the lower phase was collected and evaporated in a rotary evaporator (Büchi B-480, Uster, Switzerland) at 40 °C under 10 mbar until a constant weight was achieved. These determinations were carried out in duplicate for each sample. 

#### 3.2.4. Quality and Oxidation Indexes

The overall quality indices of the olive oils were determined by following the methodology described in the Commission Regulation (EEC) Nº 2022/2105 [41], which defines the methods of analysis to be used to assess the conformity of the olive oil with its declared category. Acidity value (AV) was measured and expressed as a percentage of free oleic acid. Oxidation indices of the oils, peroxide value (PV), expressed as meq O_2_ kg^−1^ and p-anisidine value (AnV) were measured as primary and secondary oxidation indicators, respectively. These indicators were measured based on colorimetric reactions using an Oxitester (CDR FoodLabFat, Florence, Italy) following the manufacturer’s instructions according to the official methods of AOCS, namely methods Cd 8–53 and Cd 18–90 [52], using the measurement of the absorbance at 505 nm and 366 nm for PV and AnV, respectively. These determinations were carried out in duplicate to evaluate the initial status of the oils and the status after three months of storage in amber glass vials under an inert atmosphere (N_2_) at room temperature.

In addition, the total oxidative deterioration was evaluated by calculating the TOTOX value according to

TOTOX = 2 × PV + AnV(4)

#### 3.2.5. Determination of Fatty Acid Profile

The oils studied were transformed into the corresponding fatty acid methyl esters (FAMEs) by using the BF3 derivatization method, AOAC 996.06 [53]. For that purpose, 50 mg of the sample was dissolved in 1 mL of n-hexane. Then, 1 mL of 0.5 M NaOH in methanol was added for sample methanolysis, followed by stirring for 1 min, heating at 100 °C for 10 min, and further methylation by adding 1 mL of 14% BF3 in methanol (Supelco, Pasadena, CA, USA), stirring for 1 min, and incubating at 100 °C for 10 min. A total of 1.5 mL of distilled water was added and the resulting mixture of FAMEs was extracted using n-hexane (1 mL) after stirring for 2 min at room temperature. The decanted hexane phase was then allowed to stand for 2 h with anhydrous sodium sulfate (Panreac, Barcelona, Spain) for humidity elimination. Finally, hexane was evaporated until dryness at 40 °C under a nitrogen stream by using a Stuart Block Heater SBH200D/3 (Staffordshire, UK) to obtain a residue of FAMEs, which was then dissolved in n-hexane at a final concentration of 15 mg mL^−1^. Chromatographic analysis of FAMEs was performed using a gas chromatograph Agilent Technologies (6850 N Network GC System) equipped with a flame ionization detector (FID). A sample of 1 µL was injected into the split/splitless mode (split ratio 10:1) in a capillary column HP-88 (88%-cyanopropyl) aryl-polysiloxane, length 30 m, internal diameter 0.25 mm, thickness 0.20 µm; Agilent Technologies Inc., Santa Clara, CA, USA). Helium was used as carrier gas at a flow rate of 0.9 mL min^−1^. Injector and detector temperatures were 220 and 250 °C, respectively. The temperature program was as follows: starting at 50 °C and then heating to 180 °C at 20 °C min^−1^, followed by heating from 180 to 220 °C at 15 °C min^−1^. The final temperature (220 °C) was held for 10 min. Identification of the various free fatty acids was based on a PUFA no. 3 standard obtained from Sigma-Aldrich (Steinheim, Germany). These determinations were carried out in duplicate for each sample.

#### 3.2.6. Lipid Characterization by Gas Chromatography

Gas chromatography (GC) analyses of oils were carried out in a gas chromatograph Agilent 7820A (Agilent Technologies, Santa Clara, CA, USA) with on-column injection coupled with an FID detector. The separation was performed using an HP-5MS capillary column, 5% phenyl methyl silicone (length 7 m, internal diameter 0.25 mm, and thickness 0.25 µm). The injection volume was 0.1 µL. The temperatures of the injector and detector were 50 and 340 °C, respectively. The program of temperatures started at 60 °C, increasing at 42 °C min^−1^ until 250 °C was reached. This temperature was maintained for 20 min and then increased up to 340 °C at 25 °C min^−1^, which was maintained for 35 min. The identification of the lipid classes, free fatty acids (FFA), triacylglycerols (TAG), diacylglycerols (DAG), monoacylglycerol (MAG), and minor compounds (squalene, tocopherols, and sterols) were carried out by comparing their retention times (tR) with those of different standards (oleic acid, monoolein, 1,3 diolein, squalene, tocopherols, and sterols). Once the different compounds were identified, the quantitative analysis was carried out using the external standard method [54]. For that, calibration curves with each standard from 0.05 to 6 mg mL^−1^ were prepared and analyzed. Quantitative data were expressed as a percentage of lipid classes.

#### 3.2.7. Determination of Total Phenolic Content

The total phenolic content (TPC) of the obtained oils was determined using the Folin–Ciocalteu method [55]. The method was adapted for oils as follows: 2.5 g of oil was dissolved in 5 mL of hexane, and the phenolic compounds were extracted using 3 mL of MeOH:H_2_O (60:40, *v*/*v*) for 1 min in a vortex system. Both phases were separated via centrifugation (at 3500 rpm for 10 min), and the lower phase was reextracted using 3 mL of MeOH:H_2_O (60:40, *v*/*v*) following the same procedure. The methanolic extracts were pooled and an aliquot of 10 µL was taken to an Eppendorf vial and 0.6 mL of Milli-Q water and 50 µL of Folin–Ciocalteu reagent were added. After 3 min, 0.15 mL sodium carbonate solution (20%, *w*/*v*) was added to the reaction mixture, which was finally mixed and diluted with 1 mL of. The absorbance of the solution was measured after 2 h against a blank sample using a TECAN M200 spectrophotometer at a wavelength of 760 nm. The calibration curve was constructed using standard solutions of hydroxytyrosol (HT) within the range of 0–1 mg L^−1^. These determinations were carried out in duplicate for each sample.

#### 3.2.8. Rancimat Test

The oxidation induction time or oxidative stability index (OSI) was measured at 120 °C using the Rancimat method [56], which consists of an accelerated method for the determination of the oxidative stability of the oils (Cd 12b-92) [57]. A total of 3 g of oil was weighed in a Rancimat tube and placed in an electric heating block (Metrohm 743 Rancimat stability equipment, Herisau, Switzerland). Effluent air containing volatile organic acids from the oil sample was collected in a measuring vessel containing distilled water (60 mL). The airflow was fixed at 15 L/h. The conductivity of water was measured automatically as oxidation proceeded. The Rancimat test consisted of subjecting the olive oil to forced oxidation at 120 °C until its maximum oxidation, measuring the time needed for an abrupt change in conductivity. OSI values for the samples were automatically registered as the proper endpoint. OSI values were obtained in each treatment as the average of two measurements.

#### 3.2.9. Statistical Analysis

Data analysis was performed using Excel (Microsoft Office 365) and all statistical evaluations were performed using Origin (version 9.0 for Windows; OriginLab Corporation, Northampton, MA, USA). Experiments were carried out in duplicate, and the data were expressed as mean ± standard deviation. The statistical significance of the differences between the groups was measured using one-way analysis of variance (ANOVA) and post hoc Tukey HSD test. Statistical significance was defined at the level of *p* < 0.05. 

## 4. Conclusions

This study compares the conventional extraction process with different water contents versus an alternative process based on dehydration and expeller extraction. Water reduction eliminates by-product generation but does not improve extraction yields in conventional processes. However, the dehydration of olives followed by expeller extraction yielded oils with excellent quality indexes (PV and AnV) within the category of extra virgin olive oil (less than 1% of free oleic acid and less than 20 meq O_2_/kg oil). In addition, the dehydration process does not affect the fatty acid composition. On the contrary, lower TAG content and higher DAG and free fatty acid contents were observed in the most hydrated oils. Regarding phenolic compound content and oxidative stability index, values of up to 4 and 4.7 times higher than those of oils obtained from crude olives, respectively, were found. Overall, the dehydration of olives and its combination with an expeller press for extraction of olive oil, is an excellent methodology from a holistic point of view, considering, quality, stability, healthy and nutritional properties, environmental impact, and economic value. Therefore, it should be industrially implemented as an alternative to conventional and excessively traditional extraction methodologies.

## Figures and Tables

**Table 1:** The moisture content of the olives (%), processing moisture (%), total fat content (%), extraction yield (%), oil extractability (%), and material balance (%) parameters of the oils obtained through hydraulic press conventional (CE), centrifugation (OO and OO_WW_), and expeller (dOO_1–3_) extraction systems.

Percentage (%)	CE *	OO	OO_WW_	dOO_1_	dOO_2_	dOO_3_
Initial moisture content	33–62	56.7 ± 1.3	56.7 ± 1.3	56.7 ± 1.3	56.7 ± 1.3	56.7 ± 1.3
Processing moisture	53–72	61.7	56.7	1.7 ± 0.2	6.30 ± 0.52	20.4 ± 1.45
Total fat content	20–25	21.5 ± 2.6	21.5 ± 2.6	60.7 ± 0.13	50.8 ± 1.87	33.7 ± 3.17
Extraction Yield	15–17	13.8	12.7	34.4 ± 0.25	17.6 ± 0.8	7.8
Oil recovery	60–85	59.3	57.4	57.2 ± 2.31	35.3 ± 1.06	23.3
Olive pomace ^†^ (CE, OO, OO_ww_)	30–40 ^a^	44 ^b^	52 ^b^	-	-	-
Press cake ^†^ (dOO_1–3_)	-	-	-	62.6 ± 1.3 ^c^	78.9 ± 3.6 ^d^	76.5 ^e^
OMWW	40–60	40.7	33.6	0	0	-
Material Balance	90–100	98.5	98.3	97 ± 1.29	94.1 ± 1.29	84.4 **

* [26,27]. ** Solid material could not be completely recovered from the expeller. ^†^ Moisture content of olive pomace/press cake: (^a^) 30%; (^b^) 38–43%; (^c^) 1–2%; (^d^) 5%; (^e^) 15–20%.

**Table 2:** Fatty acid profile of control and oils obtained via hydraulic press conventional (CE), centrifugation (OO and OO_ww_), and expeller extraction systems (dOO_1–3_). Significant differences (*p* < 0.05) are indicated in the same row using different letters.

(g/100 g)	CE	OO	OO_ww_	dOO_1_	dOO_2_	dOO_3_	EVOO *
Palmitic acid (C16:0)	11.5 ± 0.01 ^c^	8.16 ± 0.02 ^a^	8.16 ± 0.12 ^a^	9.84 ± 0.25 ^b^	11.8 ± 0.17 ^c^	11.06 ± 0.03 ^c^	7.00–20.0
Stearic acid (C18:0)	2.83 ± 0.01 ^c^	2.44 ± 0.02 ^b^	2.45 ± 0.02 ^b^	2.26 ± 0.00 ^a^	2.84 ± 0.01 ^c^	2.71 ± 0.02 ^d^	0.50–5.00
Oleic acid (C18:1)	71.3 ± 0.01 ^a^	77.6 ± 0.03 ^b^	77.7 ± 0.02 ^b^	77.6 ± 0.22 ^b^	76.1 ± 0.06 ^b^	76.5 ± 0.13 ^b^	55.0–85.0
Linoleic acid (C18:2)	11.5 ± 0.02 ^d^	9.30 ± 0.04 ^c^	9.42 ± 0.08 ^c^	7.28 ± 0.06 ^b^	6.00 ± 0.01 ^a^	6.01 ± 0.02 ^a^	2.50–21.0
γ-Linolenic acid (C18:3)	0.29 ± 0.01 ^a^	0.33 ± 0.02 ^a^	0.33 ± 0.01 ^a^	0.32 ± 0.01 ^a^	0.42 ± 0.01 ^b^	0.41 ± 0.01 ^b^	≤1.00
α-Linolenic acid (C18:3)	0.78 ± 0.01 ^a^	1.34 ± 0.04 ^c^	1.25 ± 0.05 ^c^	1.35 ± 0.01 ^c^	0.91 ± 0.03 ^b^	0.95 ± 0.01 ^b^	≤1.5
Others	1.80 ± 0.03 ^b^	1.91 ± 0.18 ^b^	1.53 ± 0.13 ^ab^	1.39 ± 0.03 ^a^	2.29 ± 0.03 ^c^	2.37 ± 0.11 ^c^	
Total saturated FA	14.8 ± 0.01 ^b^	10.7 ± 0.00 ^a^	10.6 ± 0.09 ^a^	12.3 ± 0.26 ^b^	14.5 ± 0.16 ^b^	14.04 ± 0.11 ^b^	
Total MUFA	72.0 ± 0.01 ^a^	78.3 ± 0.03 ^b^	78.4 ± 0.04 ^b^	77.4 ± 0.21 ^b^	77.3 ± 0.07 ^b^	77.8 ± 0.05 ^b^	
Total PUFA	12.6 ± 0.02 ^c^	10.9 ± 0.02 ^b^	11 ± 0.05 ^b^	10.3 ± 0.06 ^b^	7.19 ± 0.09 ^a^	7.20 ± 0.06 ^a^	
O/L ratio	6.2 ± 0.00 ^a^	8.35 ± 0.03 ^b^	8.25 ± 0.07 ^b^	10.7 ± 0.04 ^c^	15.2 ± 0.33 ^d^	15.3 ± 0.05 ^d^	

O/L ratio: oleic/linoleic acid ratio. * EVOO: extra virgin olive oil quality criteria, value limits set by European Union. FA: fatty acid. MUFA: monounsaturated fatty acids. PUFA: polyunsaturated fatty acids.

## Data Availability

The data obtained for the publication of this article are available upon reasonable request to the corresponding author.

## Sample Availability

Samples of all compounds are available from the authors.

## Abbreviations

OO: olive oil. dOO: dehydrated olive oil. OMWW: olive mill wastewaters. AV: acidity value. PV: peroxide value. AnV: p-anisidine value. TOTOX: total oxidative value. TPC: total phenolic content.
